# Insights on the Pooled Prevalence and Global Distribution of Leptospirosis in Goats: Systematic Review and Meta-Analysis

**DOI:** 10.3390/microorganisms12122391

**Published:** 2024-11-22

**Authors:** Roseane de Araújo Portela, Clécio Henrique Limeira, José Artur Brilhante Bezerra, Camila Marinelli Martins, Diego Figueiredo da Costa, Carolina de Sousa Américo Batista Santos, Clebert José Alves, Sérgio Santos de Azevedo

**Affiliations:** 1 Postgraduate Program in Animal Science and Health, Federal University of Campina Grande (UFCG), Av. Universitária, s/n, Patos 58708-110, PB, Brazil; roseane.portela@ifpb.edu.br (R.d.A.P.); cleciolimeira@hotmail.com (C.H.L.); artur_brilhante@hotmail.com (J.A.B.B.); carolamerico@yahoo.com.br (C.d.S.A.B.S.); clebertja@uol.com.br (C.J.A.); 2 Department of Medicine, State University of Ponta Grossa (UEPG), Av. General Carlos Cavalcanti 4748, Ponta Grossa 84030-900, PR, Brazil; camimarinelli@gmail.com; 3 Veterinary University Hospital, Federal University of Paraíba (UFPB), Campus II, Areia 58397-000, PB, Brazil; diegoveter@hotmail.com

**Keywords:** small ruminants, diagnosis, *Leptospira* spp., MAT, serogroup, serovar

## Abstract

*Leptospira* spp. infection in small ruminants is usually asymptomatic or presents nonspecific clinical signs and has an economic impact on goat farming and public health. The aim of this study was to carry out a systematic review with meta-analysis on the global seroprevalence and distribution of leptospirosis in goats. The methodology was based on the recommendations of Preferred Reporting Items for Systematic Reviews and Meta-Analyses (PRISMA), and the review project was registered in the International Prospective Register of Systematic Reviews (PROSPERO—ID: CRD42023405693). Overall, 79 articles were included in the study. The global prevalence of leptospirosis in goats was 18.6% (CI 95% = 15.08–22.71%), with no publication bias and high heterogeneity. The records originated from South America (n = 32), Asia (n = 26), Europe (n = 8), North America (n = 7), and Africa (n = 6), and seropositivities were 17%, 19%, 12%, 34%, and 22%, respectively. It is concluded that *Leptospira* spp. infection is widespread in goat herds, including regions with semi-arid and arid climates, and it is suggested that the prospects for controlling the disease should focus on avoiding environmental contamination and improving management measures and sanitary practices. This important information provides guidance for actions to protect against human contamination and to control animal leptospirosis.

## 1. Introduction

Leptospirosis is a neglected, re-emerging, and widespread zoonotic disease in developed and developing countries, common in tropical, subtropical, and temperate regions [1]. As in other animals, *Leptospira* spp. may be shed through the urine of sheep and goats and transmitted by direct or indirect contact with other animal species and humans [2]. Recent studies have reported that goats may play an important role in leptospirosis transmission as animals without clinical signs that are carriers of *Leptospira* spp. [2]. Moreover, although deemed an incidental strain, *L. noguchhi* was involved in an abortion outbreak in goats [3]. In small ruminants, *Leptospira* spp. infection is usually asymptomatic or present nonspecific clinical signs [4]; however, it negatively impairs productivity in small ruminants in different countries [5].

In goats, venereal transmission has been highlighted as a possible alternative transmission route, and the bacterium is maintained in the organs and fluids of the genital tracts of males and females [6,7]. Animals infected with adapted serovars become chronic and apparently healthy carriers, representing a source of infection for animal owners, agricultural workers, and other professionals [8,9].

The microscopic agglutination test (MAT) is deemed the reference serological diagnostic method and is mainly used as a herd test, with a cut-off point of 1:100 [10,11]. However, infected animals may be seronegative while spreading leptospires in the environment [10]. The antigens representative of circulating serogroups in a given region should be used, and when the reaction is negative, the possibility of infection cannot be ruled out [12]. Due to the very low sensitivity of MAT in chronic infections, the polymerase chain reaction (PCR) should be considered an increasingly useful tool for diagnosis in livestock to identify *Leptospira* spp. carriers [13].

There is a wide variety of *Leptospira* spp. serogroups identified in goats worldwide: Icterohaemorrhagiae [14,15,16], Pyrogenes [6], Australis [17], Panama [18], Sejroe [19], and Autumnalis [9]. Despite the broad number of papers published on goat leptospirosis, there is still no systematic review with or without meta-analysis published on the pooled seropositivity of *Leptospira* spp. infection in goats, which could be important for gathering data regarding the *Leptospira* spp. epidemiology in goats. Therefore, the objective of this study was to carry out a systematic review with meta-analysis on the seroprevalence of leptospirosis in goats and its distribution. Furthermore, a compilation of data was presented, covering the different MAT methodologies, risk factors, and circulating serogroups.

## 2. Materials and Methods

### 2.1. Survey Design

The survey consisted of a systematic review with meta-analysis based on the recommendations of Preferred Reporting Items for Systematic Reviews and Meta-Analyses (PRISMA) [20]. The review project was registered in the International Prospective Register of Systematic Reviews (PROSPERO) (https://www.crd.york.ac.uk/prospero/) (accessed on 10 January 2024), with the approval number CRD42023405693.

### 2.2. Article Search Strategy

The search was conducted in four databases between October 1st and 20th, 2023. The keywords that retrieved the largest number of articles on leptospirosis in goats were “LEPTOSPIR*” AND “GOAT”, applied in three databases: PubMed, Scielo, and Scopus. In ScienceDirect, the keywords “LEPTOSPIRA” AND “GOAT” were used. The searches were carried out without restrictions of language and year. The identified studies were sent to the ZOTERO^®^ software “https://www.zotero.org/ (accessed on 23 October 2023)”; then, duplicates, book chapters, and conference reports identified by an automatic tool were checked and excluded.

Another search method was used: the articles cited in the literature were searched individually. After checking the inclusion criteria, these articles were added to the total number of articles. The visualization of the search process was based on the PRISMA flowchart model.

### 2.3. Inclusion and Exclusion Criteria

The deemed inclusion criteria were: (i) peer-reviewed articles on cross-sectional study methodologies; (ii) articles reporting seropositivity for leptospirosis in goats by using the microscopic agglutination test (MAT); and (iii) animal-level results. After reading the title and abstract, records using polymerase chain reaction (PCR), enzyme-linked immunosorbent assay (ELISA), and latex agglutination test (LAT) techniques without association with MAT, surveys with wild goats, and reviews and technical reports were excluded. The full texts were assessed, and the reasons for exclusion were: (i) seroprevalence results per herd; (ii) use of PCR only; (iii) review of serological studies; and (iv) duplicate data with the total number of animals, seropositives, and region. Discrepant cases were resolved by consensus of two authors.

### 2.4. Risk of Bias Assessment

The methodological quality of the articles was checked using the tool provided by the Joanna Briggs Institute (JBI), applied to the prevalence [21]. After adapting the tool, excluding one criterion that applied exclusively to humans, eight criteria were applied to the studies included in the review. The total quality score ranged from 0 to 8, with one point for each ‘Yes’ received in each criterion. The studies were then classified as high, moderate, and low risk of bias, according to average scores of 0–3, 4–6, and 7–8, respectively [22]. The assessment was carried out by two authors. Moreover, the online tool Risk-Of-Bias VISualisation (RoB version 2) “https://www.riskofbias.info/welcome/robvis-visualization-tool (accessed on 29 October 2023)” was used to generate the bar graph with the frequency for each classification [23].

### 2.5. Data Extraction

The information collected from each article included: author and year; continent and geographical region; study characteristics; presence of sample calculation; leptospirosis vaccination status; sample size of goats; percentage of seropositive animals in the MAT; number of antigens used in the MAT; antibody titer cut-off point used in the MAT; and prevalent serovar and prevalent serogroup. The information contained in the summary of characteristics included: origin of the animals (live animal fair, slaughterhouse, apparently healthy, and history of reproductive disorders); location; and respective climate, according to the Köppen–Geiger climate classification.

### 2.6. Data Analysis

The inverse variance random effects model was applied to the quantitative data to estimate the pooled seroprevalence of goat leptospirosis. The presence of heterogeneity among studies was checked using Cochran’s Q test and classified using Higgins and Thompson’s *I*^2^ test. The presence of publication bias was analyzed using the inverted funnel plot and Egger’s test. Subsequently, it was necessary to complement the analyses by applying the random effect model by subgroups in the following modalities: continent, presence of sample calculation with random selection, sample size (quartiles), number of antigens, antibody titer cut-off point used in the MAT, and risk of bias. The analyses were carried out using the ‘META’ meta-analysis package run in the R environment [24], version 4.4.2, RStudio interface. A map was created using QGIS software version 3.40.0 to illustrate the number and distribution of studies according to geographical region and prevalent serogroups. To assess the risk factors for leptospirosis in goats, the online word cloud manager ‘wordArt.com’ was used. The word cloud was generated with statistically significant risk factors of the articles included in the systematic review.

## 3. Results

**Figure 1:**
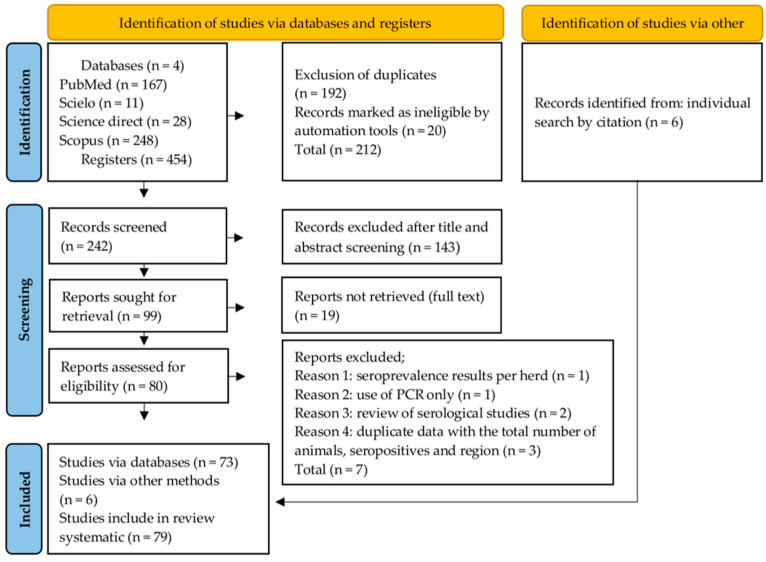
Flow diagram of the selection process for each phase and total records included in the systematic review on the global prevalence of leptospirosis in goats.

**Figure 2:**
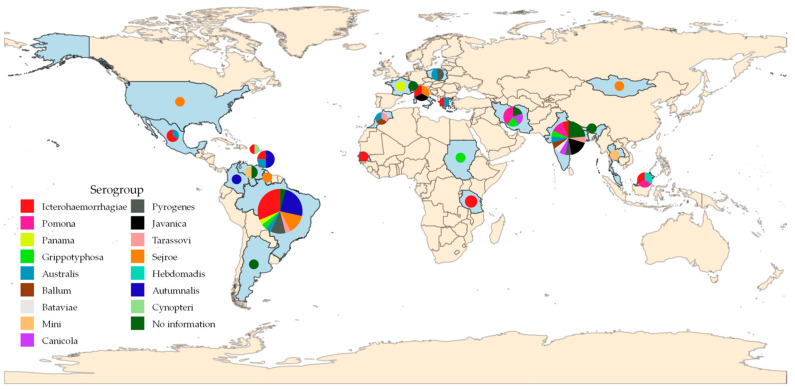
World map with the number of articles proportional to the circumference of the pie and frequency of prevalent serogroups (slices of the pie) from the articles included in the systematic review on the global prevalence of leptospirosis in goats.

The search process in the four databases retrieved 454 records. After screening, 79 articles met all the criteria, 73 through the database and 6 through individual searches (Figure 1). The records originated from South America (n = 32), Asia (n = 26), Europe (n = 8), North America (n = 7), and Africa (n = 6). The highest number of records was found in Brazil (n = 26), followed by India (n = 15), Iran (n = 5), Caribbean Islands (n = 4), Italy (n = 3), Malaysia (n = 3), Venezuela, Poland, Mexico, Morocco, and Tanzania, with two articles from each country and one article in the other countries marked on the map (Figure 2).

**Figure 3:**
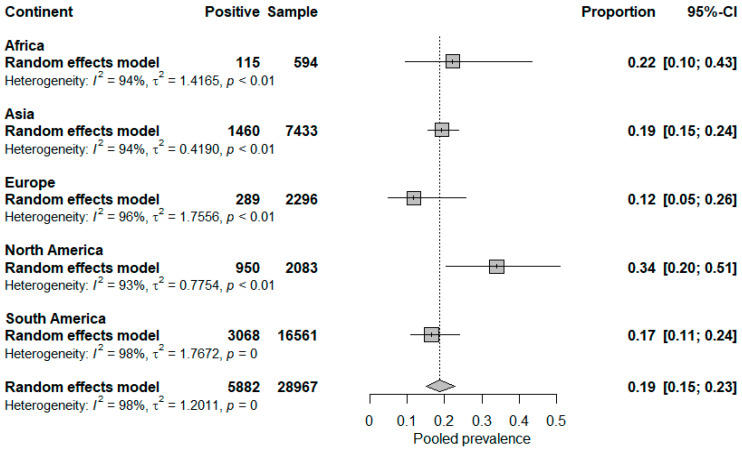
Forest plot with meta-analysis by continent subgroup included in the systematic review on the global prevalence of leptospirosis in goats.

North America presented a seroprevalence of 34% (95% CI = 20–51%), followed by Africa with 22% (95% CI = 10–43%), Asia with 19% (95% CI = 15–24%), South America with 17% (95% CI = 11–24%), and Europe with 12% (95% CI = 5–26%). Although the prevalence was higher in North America, there was no statistical difference, as observed in the forest plot of the meta-analysis by continent (Figure 3).

Goat leptospirosis studies were carried out in areas with tropical, subtropical, temperate, continental, mediterranean, semi-arid, and arid climates. Most reports involved tropical climates (n = 39; 49.4%), followed by semi-arid climates (n = 22; 27.8%), temperate climates (n = 9; 11.4%), arid climates (n = 6; 7.6%), continental climates (n = 2; 2.5%), and Mediterranean climates (n = 1; 1.3%). The ‘non-vaccination against leptospirosis’ status of the animals used was reported in 32% (25/79) of the studies (Table S1).

The prevalent *Leptospira* spp. in goats worldwide involved 16 serogroups: Icterohaemorrhagiae (n = 21 studies; 26.6%); Autumnalis (n = 11; 13.9%), Sejroe (n = 8; 10.1%), Australis (n = 7; 8.9%), Pomona (n = 5; 6.3%), Pyrogenes (n = 5; 6. 3%), Javanica (n = 4; 5.1%), Grippotyphosa (n = 4; 5.1%), Tarassovi (n = 3; 3.8%), Canicola (n = 2; 2.5%), Ballum (n = 2; 2. 5%), Panama (n = 2; 2.5%), Mini (n = 2; 2.5%), Bataviae (n = 1; 1.3%), Cynopteri (n = 1; 1.3%), and Hebdomadis (n = 1; 1.3%). However, in order to report the 16 serogroups above, it was necessary to identify the serogroup corresponding to the serovar explained in 54.4% (43/79) of the articles. For articles that did not indicate the serovar, the serogroups were identified as ‘No information’ on the map (Figure 2).

**Figure 4:**
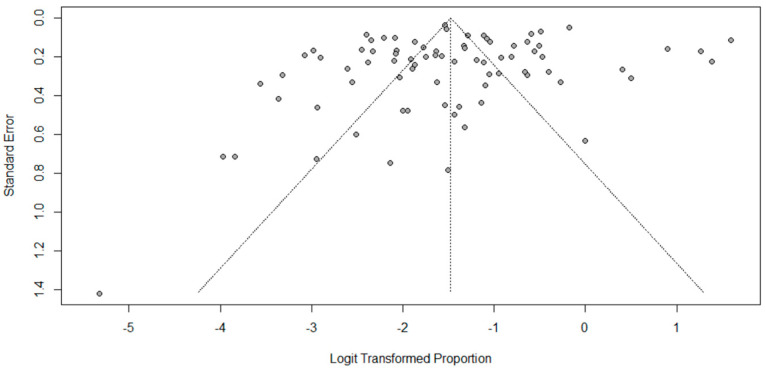
Funnel plot with the seroprevalences of the individual articles (dots) included in the systematic review on the global prevalence of leptospirosis in goats.

Pooled seroprevalence was 18.6% (95% CI = 15.1–22.7%), with the presence of heterogeneity identified by Cochran’s Q test (*p* < 0.05) and classified as high by the Higgins and Thompson test (*I*^2^ = 97.6%). The funnel plot indicated asymmetry (Figure 4), and Egger’s test applied to the asymmetric funnel plot did not indicate publication bias (*p* = 0.18). The meta-analyses of seroprevalence by subgroups showed no statistical differences but showed high heterogeneity. The deemed subgroups were: presence of sample calculation with random selection, sample size (quartiles), number of antigens used in the MAT (categories: ≤10 antigens/11 to 24 antigens/≥25 antigens), antibody titer cut-off point used in the MAT, and risk of study bias.

**Figure 5:**
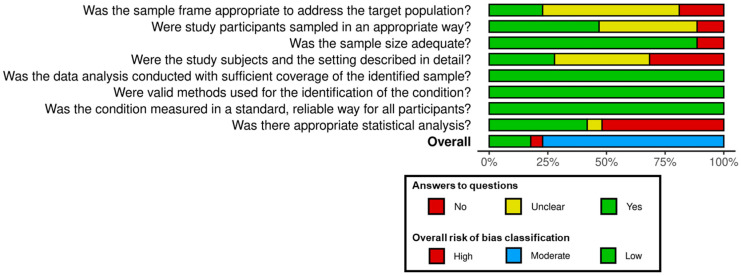
Summary plot of the risk of bias assessment of the 79 studies included in the systematic review on the global prevalence of leptospirosis in goats.

The percentage of seropositivity worldwide ranged from 0% in Brazil [25] to 83.1% in Venezuela [26]. The sample size ranged from 11 [27] to 4718 goats [9]. Regarding the number of antigens used in the MAT, the minimum was five [14], and the maximum was thirty-two [28]. When assessing the risk of bias using the JBI tool https://jbi.global/critical-appraisal-tools (accessed on 29 October 2023), 61 articles (77.2%) were considered to have a moderate risk of bias, 14 (17.7%) a low risk of bias, and 4 (5.1%) a high risk of bias (Figure 5). Information on the assignment of scores to the 79 included studies is shown in the Supplementary Material (Table S2).

Fourteen studies analyzed risk factors for goat leptospirosis, with ten from Brazil, two from Iran, one from Colombia, and one from India. However, 12 articles were analyzed; Maleki et al. [29] reported risk factors for leptospirosis generalizing different ruminant species, without specifying the goat species, and Araújo Neto et al. [30] found no statistically significant risk factor. Overall, 35 risk factors for goat leptospirosis were considered (Table 1).

**Figure 6:**
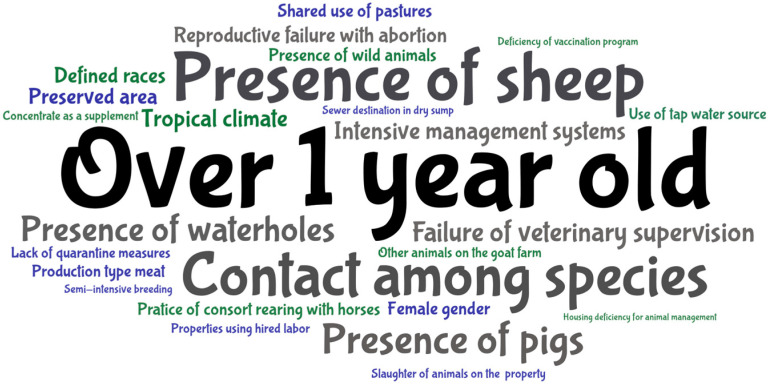
Word cloud of risk factors for leptospirosis in goats included in the systematic review on the global prevalence of leptospirosis in goats.

Risk factors that had similar meanings were combined to be counted with the same keyword frequency (i.e., ‘Ingestion of still water’ and ‘Presence of waterholes’ = ‘Presence of waterholes’; ‘1 to 3 years old’; and ‘1.5 to 4 years old’ = “Over 1 year old”). After adjustments, 26 keywords were included in the word cloud, and the highest frequency was for the risk factor ‘Over 1 year old’, with three occurrences. ‘Contact among species’, ‘Intensive system production’, ‘Presence of waterholes’, ‘Failure of veterinary supervision’, ‘Presence of sheep’, ‘Reproductive failure with abortion’, and ‘Presence of pigs’ had two occurrences each, and the others had one occurrence (Figure 6).

## 4. Discussion

The evidence found in this survey indicate a scenario in which *Leptospira* spp. infection in goats is spread worldwide. The highest concentration of publications was in South America, with 16,561 samples tested. In Venezuela, the prevalences were high (83.1% and 77.87%) [26,43], and low prevalences were observed in Argentina (7.2%), Guyana (8.9%), and Trinidad Island (3.3%) [44,45,46]. In Brazil, the prevalence ranged from 0% [25] to 80% [47]. The highest representation of studies was in Brazil (n = 26), due in part to the country having a well-developed dairy goat farming sector [48], in addition to publications from animal leptospirosis research groups in northeastern and southeastern Brazil.

Seroprevalence on the Asian continent ranged from 4.4% in Malaysia [49] to 50% in Mongolia [19]. India had the largest number of studies (n = 15), reflecting the importance of the disease, which is endemic to goats and sheep in the region [50]. In addition to India, other countries that had a high number of goats, such as China and Bangladesh [51], had no reports of seroprevalence. Therefore, the Asian continent, which holds 52% of the world’s goat farming [51], has a limited number of studies on leptospirosis in goats. The importance of serological studies conducted in specific areas and the impact of the infection on the health and production of small ruminants, in addition to their role as hosts or accidental disseminators, were highlighted [50].

In North America, seroprevalence ranged from 11% in St Croix [15] to 71.1% in Mexico [52]. The high prevalence was associated with infection, since the animals were not vaccinated against leptospirosis in Mexico [52]. In the United States of America, the unavailability of vaccination data was relevant to interpreting seroreactivity, where exposure is not indicative of infection [53], as the MAT does not differentiate between an antibody response from natural infection and a vaccine response [10,53]. Only 32% of the studies mentioned the status of ‘not vaccinated against leptospirosis’, suggesting that vaccination against leptospirosis in goat herds worldwide is not practiced [45,54,55]. Vaccination should be encouraged in regions with higher prevalence in an attempt to reduce reproductive losses [56].

In Europe, the lowest prevalences were observed in Italy, from 2.1% to 4.8% [57,58]. A prevalence of 60% was reported in a study that tested 60 goats destined for slaughter on Reunion Island, located in the Indian Ocean but belonging to France [18]. Globally, this was the highest seroprevalence found using samples from goats destined for slaughter. In the few studies in the world involving goats exclusively from slaughterhouses, the variation was between 5% and 24.8% [7,54]. It is important to emphasize the circulation of infected animals without clinical signs, which represents a great risk to human health, as demonstrated in the results involving goats from slaughterhouses and live animal fairs [6,7,15,18,26,34,54,59].

There have been few studies in Africa (n = 6), with only 594 goats tested from four countries on a continent that holds 39% of the goat population [51]. It is necessary to obtain information on the impact of this disease, which is highly neglected in Africa [14]. However, high prevalence rates have been reported, higher than the global average, with 62% seroprevalence in Tanzania [14], 34.6% in Senegal [16], and 30% in Sudan [60]. Therefore, given the dynamism of leptospirosis, infection in goats is present in climatic regions that were not previously associated with the presence of the disease. Leptospires are becoming increasingly adapted, finding alternative routes of survival and transmission to escape adverse environmental conditions [6,9]. In addition to the African countries, high prevalences in semi-arid conditions have also been reported in Brazil [61], indicating the exposure of goats to *Leptospira* spp. in environmental conditions adverse to the survival of the bacteria.

The Icterohaemorrhagiae serogroup was the most frequent in goats worldwide, particularly in Brazil, Mexico, Tanzania, and Senegal. This reflects the global trend as the serogroup most involved in animal and human infections [62,63]. The Autumnalis serogroup was the second most frequent in goats and was predominant in Colombia and Barbados Island. In Colombia, it was the most frequent serogroup in sheep [32], and in Barbados Island, rodents and cattle [64]. It is likely that this serogroup has adapted to small ruminants in regions of Brazil [9,65]. The Autumnalis serogroup has been shown to represent a risk of infection for humans and other animals, as well as pointing to rodents as the main reservoir [66,67].

The Sejroe serogroup was predominant in the United States of America, with high prevalence in wild animals [53], and in Mongolia, in sheep and camels [19]. For the Hardjo serovar, whose main reservoir is cattle [10], it is suggested that infection in goats depends on the possibilities of contact with the agent in the natural environment in the presence of cattle [42,68]. The Australis serogroup was the fourth most frequent in the world. In the United States of America, Australis was the most frequent in cattle, pigs, and exotic animals [53], and in Europe, the Bratislava serogroup was adapted to horses [69]. Ratifying the interspecies relationship, the seropositivity found was indicative of interspecies transmission [9].

The Pomona serogroup was predominant and reported exclusively in Asia, most frequently in Iran in animals destined for slaughter [70] and in animals with reproductive problems [71]. It is important to highlight the experimental resistance of this serogroup in sheep in New Zealand and Brazil [72,73]. The Pyrogenes serogroup was reported in Brazil and India as an indication of the involvement of rodents as a possible source of infection for goats [6,7,74]. The Pyrogenes serovar has wild animals as reservoirs, which can be a source of infection and can spread the agent to herds [36]. The Javanica serogroup was the most common in India and had a high frequency in buffalo and cattle [75]. This serogroup was isolated from a wild rat [76], and high antibody titers were reported in sheep [77]. Although Italy indicates that it has the Poi serovar of the Javanica serogroup, a cross-reaction has been suggested [57].

In order to use the MAT correctly, it is essential to recognize the diversity of serogroups involved in *Leptospira* spp. infection in goats in each region, so it is imperative to include the correct serogroups in the MAT, or the results may not correspond to the potential risk in the area studied [53]. It is known that the MAT can reliably identify the presumptive serogroup, but it is not possible to identify the specific serovar, due to the high degree of cross-reactions between the different serovars in each serogroup [78,79]. Therefore, MAT results should be presented by serogroup, a recommendation that was not identified in 54.4% (43/79) of the articles in this review.

The confidence interval for the estimated prevalence of 18.6% (95% CI = 15.08–22.71%) was narrow, which allowed for a better reliability of the result [80]. Visualization of the funnel plot showed asymmetry, suggesting publication bias; however, the Egger’s test indicated no publication bias. Thus, the asymmetry observed in the funnel plot may be related to high heterogeneity. Most analyses of prevalence studies show high heterogeneity, above 90% [81], as reported by Araújo et al. [82] in a meta-analysis of the global seroprevalence of leptospirosis in pigs (*I*^2^ = 99.4%). A possible cause of the high heterogeneity is probably associated with the 77.2% of articles with a moderate risk of bias. Important sources of heterogeneity are often unknown and can be caused by differences in methodological quality [83]. The most common type of study included in this research was cross-sectional, a frequently designed observational study in veterinary epidemiology, but it can be subjected to several systematic biases, including selection bias [84]. To reduce this type of bias in observational studies, standardizing methodologies based on probabilistic sampling and random selection must be used to generate reliable results [85].

The found prevalence of 18.6% is relevant for the goat species and for health worldwide, as infected goats are sources of infection in the herd and can infect other animals and humans [34]. Furthermore, it should be noted that the prevalence may be underestimated considering the carrier condition, as reported by Soares et al. [6], due to the case of host-adapted infections where the MAT has limitations [12]. The importance of leptospirosis in goats has been reported to be greater than that of brucellosis, and it plays a major role in the etiology of reproductive problems in Venezuela, Brazil, and Mexico [43,52,68]. Few studies have addressed a causal link with reproductive problems, and recent research has identified leptospiral DNA in the organs of goat fetuses and reproductive systems of goats [6,7]. Therefore, leptospirosis should be considered when assuming a diagnosis in goats with reproductive failures [71].

In goats, the cut-off point of 1:20 used in the MAT in Asia and Africa was the same as that used for other species [14,17]. The cut-off point of 1:40 was considered the closest to that used in seroepidemiological surveys in Asian regions, which is 1:50 [74]. In South America, the 1:50 cut-off point has been suggested for greater sensitivity in detecting anti-*Leptospira* spp. antibodies in carrier animals [6,86]. Hamond et al. [87] used a cut-off point of 1:200 to minimize cross-reactions in animals from endemic regions with a history of reproductive problems. Using a cut-off point of 1:200 for samples from herds with a history of reproductive problems is not a consensus. Gaytán-Camarillo et al. [88] used a cut-off point of 1:40, while the other studies [27,42,43,71,89,90] maintained the recommended cut-off point for herds of 1:100 [11]. Therefore, the possibility of detecting an animal that maintains or disseminates *Leptospira* spp. increases with a diagnostic protocol adapted to a specific region [91].

Leptospirosis in goats depends largely on the possibility of contact with leptospires in the environment [42], and this condition is associated with the following risk factors: goats aged over 1 year old due to the longer exposure time for infection to occur [31,38,41]; other animals in the goat herd [33]; presence of sheep [35,40]; practice of consort rearing with horses [34]; presence of pigs; presence of wild animals; shared use of pastures; sewer destination in a dry sump [36]; preserved area [38]; semi-intensive breeding [37]; and presence of waterholes [39]. For the variable ‘Contact among species’, the author explained the condition of cohabitation between herds with heifers and calves during grazing or during mating [33], as well as breeding with sheep, cattle, and swine [41]. Wild and domestic animals can be kidney carriers, especially rodents, cattle, pigs, and dogs [78].

Other risk factors are associated with failures and/or absences of sanitary and management practices: intensive management systems; properties using hired labor; defined breeds [41]; failure of veterinary supervision; lack of quarantine measures; housing deficiency for animal management [32]; deficiency of vaccination programs [33]; concentrate as supplement [40]; slaughter of animals on the property; meat production type [36]; and occurrence of abortion [39]. Lilenbaum et al. [42] reported that abortion and other reproductive failures are consequences of the presence of the disease and not risk factors. The risk factors associated with the seropositivity and transmission of leptospirosis are important because they reveal data that can be used to correct flaws in management [82]. The climatic factor associated with leptospirosis in goats is considered to be the ‘tropical climate’ variable, due to heat stress affecting the condition of the animal and rainfall creating a favorable environment for the survival of leptospires [42]. The ‘female gender’ risk factor is controversial, and different results have been recorded for goat leptospirosis [31,32,33,36,37,38,40,41].

Saranaya et al. [33] pointed to the existence of different risk factors associated with the transmission of leptospirosis in two completely different epidemiological contexts in India. In Brazil, Santos et al. [38] reported on the risk factors affecting seroprevalence in goats, which are different from the risk factors for other species, including sheep. This demonstrates the relevance, for leptospirosis control, of serological surveys that analyze risk factors, as they inform about the different serogroups and the conditions associated with the higher occurrence of infection in a given region and animal species.

This study had a limitation in opting to exclude gray literature, e.g., research reports, conference papers, dissertations and theses, government documents, and other research outputs. The inclusion of gray literature could have resulted in a wider range of locations, as well as reduced the possible risk of publication bias [92]. Therefore, the lack of studies in certain continents may be due to the neglect of the disease, as previously reported [14,18], but it may also be due to the limitation presented.

## 5. Conclusions

It is concluded that *Leptospira* spp. infection is widespread in goat herds all over the world, including in regions with semi-arid and arid climates. The high level of seropositivity in animals destined for slaughter and the involvement of the Icterohaemorrhagiae serogroup raise public health concerns, since goats are involved in the transmission of leptospirosis. The prospects for controlling the disease should focus on avoiding environmental contamination and improving management measures and sanitary practices. This important information provides guidance for actions to protect against human contamination and to control animal leptospirosis.

## Figures and Tables

**Table 1:** Risk factors for leptospirosis in goats extracted from articles included in the systematic review with meta-analysis on the pooled seroprevalence of leptospirosis in goats.

Author/Country	Statistically Significant Risk Factors
[31] Iran	Female gender
	1.5 to 4 years old
[32] Colombia	Lack of quarantine measures of acquired animals
	Housing deficiency for animal management
[33] India	Use of tap water source in goat farms
	Presence of pigs in goat farms
	Contact with other animals
	Other animals on the goat farm
	Deficiency of the vaccination program
[34] Brazil	Intensive management systems
	Practice of consort rearing with horses
[35] Brazil	No veterinary services
	Sheep herd over 52 animals
[36] Brazil	Presence of wild animals such as deer and capybaras on the property
	Presence of pigs on the property
	Production-type meat
	Sewer destination in a dry sump
	Shared use of pastures
	Slaughter of pigs, sheep, cattle, and goats
	Frequent occurrence of abortion
[37] Brazil	Ingestion of still water, including supplies from buckets, wells, dams, and ponds
[38] Brazil	Age 1 to 3 years and older than 3 years
	Preserved area
[39] Brazil	Presence of waterholes
	Semi-intensive breeding
	Reproductive failure with abortion
[40] Brazil	Farms that provided concentrate as a supplement
	Presence of contact between sheep and goats
[41] Brazil	Adult goats
	Defined races
	Intensive systems of goat production
	Properties using hired labor
	Contact among species
[42] Brazil	Tropical climate
	Frequency of professional veterinary supervision

## Data Availability

The datasets generated during and/or analyzed during the current study are available from the corresponding author upon request.

## Supplementary Materials

The following supporting information can be downloaded at: https://www.mdpi.com/article/10.3390/microorganisms12122391/s1. Table S1: Data extracted from 79 studies included in a systematic review with meta-analysis on the global seroprevalence of leptospirosis in goats; Table S2: Results of the critical assessment of the methodological quality of the studies included in the systematic review with meta-analysis on the global seroprevalence of leptospirosis in goats, using the critical assessment tool for studies with JBI prevalence data. References [93,94,95,96,97,98,99,100,101,102,103,104,105,106,107,108,109,110] are cited in the Supplementary Materials.
