# Supplementary material for: Insights on the Pooled Prevalence and Global Distribution of Leptospirosis in Goats: Systematic Review and Meta-Analysis
Source: Microorganisms. 2024 Nov 22;12(12):2391. doi: 10.3390/microorganisms12122391 (PMC11676935; doi:10.3390/microorganisms12122391)
Supplement: Supplementary file 1 [file microorganisms-12-02391-s001.zip › microorganisms-3288296-table-S1.pdf]

Table S1 - Data extracted from 79 studies included in systematic review with meta-analysis on the global seroprevalence of leptospirosis in goats.

Author / year (publication)	Geographical region and continent	Characteristics of the animals, location and climate - Köppen-Geiger climate classification	Vaccination against leptospirosis	Calculation sample size	MAT					
					Sample size	Positive (%)	Cutoff point	Nº of antigens	Prevalent serovar	Prevalent serogroup
1.Anderson et al., 2023	United States North America	Goat sera tested from Missouri, Indiana and Illinois - Several climates.	NI	N	55	40	1:100	15	-	Sejroe
2.Galvão et al., 2023	Brazil South America	Goats originating from an indigenous Community, state of Pernambuco - Semiarid.	N	N	108	1.85	1:50	15	NI	NI
3.Ramin et al., 2023	Iran Asia	Goats from farms in different regions in Urmia suburbs - Semiarid.	N	N	130	12.75	1:100	6	NI	NI
4.Soares et al., 2023	Brazil South America	Blood samples collected from male goat, in the public slaugtherhouse in the state of Paraíba - Semiarid.	NI	Y	40	7.5	1:50	24	-	Pyrogenes
5.Guzman-Barragan et al., 2022	Colombia South America	Goat from 49 farm in northeastern areas of Colombia including the peninsular zone of La Guajira and Cesar departments - Semiarid.	NI	Y	245	16.2	1:100	13	Autumnalis	-
6.Soares et al., 2022	Brazil South America	Blood samples collected from female goat, in the public slaugtherhouse in the state of Paraíba - Semiarid.	NI	Y	40	5	1:50	24	-	Pyrogenes
7.Viana et al., 2022	Brazil South America	Samples of goats from different states belonging to the Northeast region - Seimarid.	NI	Y	4.718	17.7	1:100	32	-	Autumnalis and Australis
8.Rocha et al., 2022	Brazil South America	Blood samples collected from female goat, in the public slaugtherhouse in the state of Paraíba - Semiarid.	NI	N	34	17.6	1:100	24	-	Autumnalis; Ictero** and Tarassovi
9.Balamurugan et al., 2021	India Asia	Apparently healthy goats from coastal districts of Andhra Pradesh, Kerala, Tamil Nadu and Puducherry - Several climates.	NI	N	868	38	1:100	18	Bataviae	-

10.Carvalho et al., 2021	Brazil South America	Samples from dairy herds from Recôncavo Baiano, in the state of Bahia - Tropical.	NI	N	125	80	1:100	24	-	Autumnalis
11.Cranford et al., 2021	Virgin Island North America	Blood samples collected from goats in a USDA-approved abattoir, on the island of St. Croix – Tropical.	N	N	19	11	1:100	18	-	Ictero** and Cynopteri
12.Gaytán-Camarillo et al., 2021	Mexico North America	Goats from farms with a history of reproductive problems, belonging to 19 cities in the state of Guanajuato - Temperate / Semiarid.	NI	N	1.640	45.5	1:40	6	Ictero**	-
13.Maleki et al., 2021	Iran Asia	Serum samples from apparently healthy goats from regions of Lorestan - Semiarid / Arid.	N	Y	195	14.87	1:100	7	Canicola	-
14.Mgode et al., 2021	Tanzania Africa	Serum samples from 6 farms in the village of Nagulo Bahi, Bahi district – Semiarid.	NI	N	45	62	1:20	5	Sokoine	Ictero**
15.Rahman et al., 2021	Malaysia Asia	Serum samples from 10 districts of Kelantan – Tropical.	NI	N	366	11.20	1:100	19	Hebdomadis	-
16.Saranya et al., 2021	India Asia	Goats of the Northeastern and Southern Provinces - Tropical / Subtropical	NI	N	216	37.4	1:20	12	Ballum and Grippotyphosa	-
17.Alamuri et al., 2020	India Asia	Serum samples collected from 160 villages in the districts of Gujarat, Navsari and Surat - Tropical.	NI	N	146	36.3	1:100	18	Javanica	-
18.Suarez et al., 2020	Argentine South America	Goats form 35 rural properties propieti in the municipality of Payogasta, province of Salt – Arid.	NI	N	139	7.2	NI	NI	NI	NI
19.Vihol et al., 2020	India Asia	Blood samples collected from goat, in the public slaugtherhouse in Surat, Gujarat - Tropical.	N	N	42	11.90	1:40	12	Pyrogenes	-
20.Bertelloni et al., 2019	Italy Europe	Goats from farms and slaughterhouses in Tuscany - Subtropical.	NI	N	327	2.75	1:100	8	Ictero**	-
21.Dhivahar et al., 2019	India Asia	Blood samples collected from goats with reproductive problems in Kerala districts and veterinary centers - Tropical.	NI	N	62	25.81	1:100	12	Pomona	-
22.Pimenta et al., 2019	Brazil South America	Serum samples from four northeastern brazilian states - Several climates.	NI	N	1.761	8.3	1:100	27	-	Autumnalis

23.Roqueplo et al., 2019	Senegal Africa	Goats from two villages, located in Dielmo and Ndiop - Semiarid / Arid.	N	N	52	34.6	1:100	24	-	Ictero**
24.Shiokawa et al., 2019	Caribbean Island North America	Blood samples collected from goat, in the slaugtherhouse on the island of Saint Kitts – Tropical.	N	N	105	24.8	1:50	21	Bratislava	-
25.Álvarez et al., 2018	Mexico North America	Serum samples from two dairy goat units in Apaseo el Alto, Guanajuato - Temperate.	N	N	194	71.1	1:100	12	Ictero**and Bratislava	-
26.Sabarinath et al., 2018	India Asia	Blood samples from healthy and suspected leptospirosis goats of 16 India states - Several climates.	N	N	2.057	17.88	1:100	16	Ictero**	-
27.Shrestha et al., 2018	Nepal Asia	The study population consisted of goat blood samples from 239 febrile humans in Kaski - Temperate.	NI	N	181	17	1:50	20	NI	NI
28.Alves et al., 2017	Brazil South America	Goats from live animal fair in the state of Pernambuco - Semiarid.	NI	Y	233	6.87	1:100	22	Ictero**	-
29.Balamurugan et al., 2017	India Asia	Goats from 12 different districts of Odisha - Tropical / subtropical.	NI	N	120	28.33	1:100	18	Australis and Tarassovi	-
30.Campos et al., 2017	Brazil South America	Goats from 33 farms in 12 municipalities of Teresina, Piauí state - Tropical.	N	Y	292	34.6	1:100	22	Ictero**	-
31.Pasquali et al., 2017	Brazil South America	Goats from 95 properties in 18 regional centers in the state of Paraná – Tropical.	NI	N	1.055	9.9	1:100	22	Pyrogenes	-
32.Rizzo et al., 2017	Brazil South America	Apparently healthy goats from 15 municipalities in Sergipe, including three mesoregions - Tropical / Semiarid.	NI	Y	675	24.74	1:100	22	-	Ictero**
33.Santos et al., 2017	Brazil South America	Goats from rural preserved area and around the conservation unit in the Caatinga biome properties - Tropical / Semiarid.	NI	N	576	13.4	1:100	24	Ictero**	Ictero**
34.Schnydrig et al., 2017	Switzerland Europe	Goats with reproductive problems and abortions in 11 cities - Several climates.	NI	N	11	18.2	1:100	9	NI	NI
35.Vihol et al., 2017	India Asia	Apparently healthy goats from villages, slaughterhouses and College of Veterinary and Animal Husbandry, Gujarat -Tropical.	N	N	292	20.86	1:40	17	NI	NI

36.Balamurugan et al., 2016	India Asia	Serum samples collected in Andhra Pradesh, Kerala, Gujarat, Maharashtra and Tamil Nadu - Several climates.	NI	N	107	38.3	1:100	14	NI	NI
37.Benkirane et al., 2016	Marocco Africa	Apparently healthy goats from slaughterhouses, farms and vaccination sites in the country of Temara, Sidi Kacem and Oulmes - Temperate.	N	N	30	20	1:20	14	-	Australis and Ballum
38.Costa et al., 2016	Brazil South America	Blood samples collected from goats in slaughterhouses in 11 country in the state of Paraíba - Semiarid.	NI	Y	500	5.2	1:100	24	Hardjobovis	-
39.Parveen et al., 2016	India Asia	Goats from miners and arround in the Pudukkottai and Karur, districts of Tamil Nadu - Tropical.	NI	N	29	24.1	1:160	12	-	Javanica
40.Rezaei et al., 2016	Iran Asia	Blood samples collected from apparently healthy goats from 8 areas of Ahvaz, Semiarid.	N	N	210	10.95	1:100	08	Pomona	-
41.Souza et al., 2016	Brazil South America	Goats from farms with a history of reproductive problems in Uberlândia, Minas Gerais – Tropical.	N	N	19	21.05	1:100	12	Grippotyphosa	-
42.Tagliabue et al., 2016	Italy Europe	Serum samples from Italian institutes – Temperate.	NI	N	765	4.8	1:400	9	Hardjo	-
43.Vihol et al., 2016	India Asia	Apparently healthy and sick goats in villages, southern Gujarat and slaughterhouse in Surat - Tropical.	NI	N	459	25*	1:40	17	NI	NI
44.Assenga el al., 2015	Tanzania Africa	Apparently healthy goats from households in the Katavi region - Tropical.	NI	N	248	8.47	1:160	07	-	Ictero**
45.Benkirane et al., 2015	Marocco Africa	Goats from five farms with reproductive problems in two regions, Tetouan and Chaouen - Temperate.	N	N	99	5	1:100	09	Tarassovi	-
46.Cortizo et al., 2015	Brazil South America	Goats from 12 herds in the state of Espírito Santo - Tropical.	NI	Y	296	11.1	1:100	7	Ictero**	Ictero**
47.Silva et al., 2015	Brazil South America	Blood samples collected from farms in the Caatinga region, belonging Ceará and Bahia states - Semiarid.	NI	N	37	43.2*	1:100	24	Panama	-

48.Topázio et al., 2015	Brazil South America	Goats form 120 herds from 57 countries in the state of Santa Catarina – Temperate.	NI	Y	654	35.47	1:100	10	-	Ictero**
49.Abdollahpour et al., 2014	Iran Asia	Blood samples collected from apparently healthy goats in the slaugtherhouse, in the Urmia - Semiarid.	NI	N	130	19.2	1:100	6	Pomona	-
50.Albuquerque et al., 2014	Brazil South America	Goats from 13 dairy farms from different regions in the state of Bahia - Semiarid.	NI	Y	102	0	1:100	17	-	-
51.Hamond et al., 2014	Brazil South America	Serum samples from herds with reproductive problems, in the state of Rio de Janeiro - Tropical.	N	N	59	34	1:200	28	-	Ictero**
52.Paramasivan et al., 2014	India Asia	Blood samples collected from apparently healthy goats, in the slaugtherhouse in the Pudukkottai - Tropical.	NI	N	202	12.87	1:100	NI	Canicola	-
53.Crespo et al., 2013	Venezuela South America	Goats from 13 sectors of Miranda, state of Zulia - Tropical.	N	Y	550	83.1	1:100	12	Sari	-
54.Desvars et al., 2013	France Europe	Blood samples collected from apparently healthy goat, in the slaugtherhouse in the Reunion Island - Tropical.	N	N	60	60	1:100	17	-	Panama
55.Suwancharoen et al., 2013	Thailand Asia	Goats from 36 provinces - Tropical.	NI	N	516	7.9	1:50	24	Mini	-
56.Hassanpour et al., 2012	Iran Asia	Blood samples without clinical goats from 12 goat herds of Khoy - Semiarid.	N	N	150	13.30	1:100	6	Grippotyphosa	-
57.Higino et al., 2012	Brazil South America	Blood samples from 110 dairy goat herds in the state of Paraíba - Semiarid.	NI	Y	975	8.7	1:100	24	Autumnalis	-
58.Martins et al., 2012	Brazil South America	Goats herds from six regions of the state of Rio de Janeiro - Tropical.	NI	Y	343	25.9	1:100	13	Hardjo	-
59.Santos et al., 2012	Brazil South America	Goats from 11 properties in the country Uberlândia, state of Minas Gerais – Subtropical.	NI	Y	230	31.3	1:100	15	Autumnalis	-
60.Valeris-Cacin et al., 2012	Venezuela South America	Goats farms with reproductive problems in Mauroa, state of Falcón - Tropical.	N	Y	199	77.87	1:100	12	NI	NI
61.Ananina et al., 2011	Mongolia Asia	Blood samples obtained from goats from 7 regions in central, southern and eastern Mongolia - Several climates.	NI	N	10	50	1:100	13	-	Sejroe

62.Czopowicz et al., 2011	Poland Europe	Goats from various parts of Poland - Continental.	N	Y	736	21.6	1:100	19	Zanoni	Pyrogenes
63.Suepaul et al., 2011	Caribbean Island South America	Serum samples obtained from goats farms from Trinidad Island - Tropical.	N	Y	180	3.3	1:100	23	Copenhageni	Ictero**
64.Araújo Neto et al., 2010	Brazil South America	Goats from 12 herds in the state of Rio Grande do Norte - Semiarid.	NI	Y	366	14.5	1:100	22	Autumnalis	-
65.Krupakaran et al., 2009	India Asia	Serum samples collected from goats from different parts of Karur, Tamil Nadu – Tropical.	NI	N	120	23.33	1:100	NI	Pomona	-
66.Lilenbaum et al., 2008	Brazil South America	Goats from 13 farms with reproductive problems, in the state of Rio de Janeiro - Tropical.	N	Y	248	20.8	1:100	24	Hardjo	-
67.Lilenbaum et al., 2007	Brazil South America	Blood samples collected from herds goats located in the state of Rio de Janeiro - Tropical.	NI	Y	1000	11.1	1:100	12	Hardjo	-
68.Krawczyk M., 2005	Poland Europe	Goats from in the northern part, near the town of Torun - Continental.	NI	N	104	12	1:100	18	Bratislava	-
69.Sunder, et al. 2005	India Asia	Blood samples from goats different parts of the villages on the Andaman and Nicobar Islands - Tropical.	NI	N	67	16.42	NI	NI	NI	NI
70.Burriel et al., 2003	Greece Europe	Blood samples collected from farm on the Islands of Greece - Mediterranean.	N	N	198	16.2	1:100	18	Bratislava; Australis and Copenhageni	-
71.Schmidt et al., 2002	Brazil South America	Blood samples from 22 gotas farms in the state of Rio Grande do Sul - Temperate.	NI	N	345	3.4	1:100	12	Ictero**	-
72.Ciceroni et al., 2000	Italy Europe	Goats from 26 farm in Alto Adige - South Tyrol - Temperate.	NI	N	95	2.1	1:100	28	Poi	-
73. Natarajaseenivasan; Ratnam, 1999	India Asia	Blood samples collected from gotas at the Saidapet slaughterhouse in Chennai - Tropical.	NI	N	40	12	1:100	10	-	Javanica
74.Bahaman et al., 1987	Malaysia Asia	Blood samples obtained from government, agricultural goas farms and slaughterhouses - Tropical.	N	N	657	4.4	1:40	10	Pomona	-

75.Motie; Meyers, 1986	Guyana South America	Sample sérum collected from 13 farms in five regions of Guyana. Tropical.	NI	N	417	8.9	1:100	12	-	Sejroe
76.Everard et al., 1985	Caribbean Island North America	Goats from several locations in the Grenada Island - Tropical.	NI	N	44	25	1:100	24	-	Autumnalis
77.Damude et al., 1979	Caribbean Island North America	Blood samples from goats in the Barbados Island -Tropical.	NI	N	26	19	1:100	17	-	Autumnalis
78.Shigidi,1974	Sudan Africa	Sera obtained from Khartoum Central Abattoir, Khartoum Veterinary Hospital and Faculty of Veterinary Sciences Clinical Sciences Clinic - Arid.	NI	N	120	30.8	1:16	12	Grippotyphosa	-
79.Smith., et al., 1961	Malaysia Asia	Blood samples collected from goats in rice paddies, scrubland and various parts of Malaysia - Tropical.	NI	N	61	28	1:100	31	Ictero**	-

NI- No information on the goat species; N – No; Y- Yes; * percentage calculated from the absolute value of animals; ** icterohaemorrhagiae.

References

Abdollahpour, Gh., Ramin, A., Khalili, Y., 2014. Serological evaluation of *leptospira* serotypes using microscopic agglutination test in Urmia goats. J. Anim. Sci. Res. 24, 71-81. https://magiran.com/p1286571.

Alamuri, A., Veena, S., Kumar, K.V., Kalyani, I.H., Rahman, H., Shome, B.R., Balamurugan, V., 2020. Changing Trend in the Prevalence and Emergence of *Leptospira* Serogroup-Specific Antibodies in Livestock in Gujarat, India. Proc. Natl. Acad. Sci., India, Sect. B Biol. Sci. 90, 1145–1151. https://10.1007/s40011-020-01186-y.

Albuquerque, I.R.R., Gois, G.C., Campos, F.S., 2014. Profile Goat Herd Health in Senhor of Bonfim, Bahia. Act. Vet. Bras. 8, 144–149. https://pesquisa.bvsalud.org/portal/resource/pt/vti-716786.

Álvarez, M.A.L., Escatell, G.S., Arzate, J.J.M., Ibarra, J.M.O., Rivera, E.M.L., 2018. Anticuerpos contra *Leptospira* spp en caprinos lecheros en Guanajuato, México. Rev. Invest. Vet. Perú 29, 611–618. https://10.15381/rivep.v29i2.14525.

Alves, J.R.A., Lima, G.M.S., Silva, J.D., Costa, D.F., Santos, F.A., Higino, S.S.S., Azevedo, S.S., Alves, C.J., 2017. Epidemiological characterization and risk factors associated with leptospirosis and brucellosis in small ruminants sold at animal fair in the Sertão Region of Pernambuco State, a semiarid Region of Northeastern Brazil. Semina: Ciências Agrárias 38, 1933–1946. https://10.5433/1679-0359.2017v38n4p1933.

Ananina, I.V., Korenberg, E.I., Tserennorov, D., Savel'eva, O.V., Batjav, D., Otgonbaatar, D., Enkhbold, N., Tsend, E., Erdenechimeg, B., 2011. Detection of leptospirosis infection in certain wild and domestic animals in Mongolia. J. Microbiol. Epidemiol. Immunobiol. 88, 36–39. https://pubmed.ncbi.nlm.nih.gov/22145347/.

Anderson, T., Hamond, C., Haluch, A., Toot, K., Nally, J.E., LeCount, K., Schlater, L.K., 2023. Animals Exposed to *Leptospira* Serogroups Not Included in Bacterins in the United States and Puerto Rico. Trop. Med. Infect. Dis. 8, 183. https://10.3390/tropicalmed8030183.

Araújo Neto, J.O., Alves, C.J., Azevedo, S.S., Silva, M.L.C.R., Batista, C.S.A., 2010. Seroprevalence of leptospirosis in goats of the Seridó Oriental microregion, Rio Grande do Norte State, Northeastern Brazil, and risk factors research. Braz. J. Vet. Res. Anim. Sci. 47, 150-155. https://www.revistas.usp.br/bjvras/article/view/26839/28622.

Assenga, J.A., Matemba, L.E., Muller, S.K., Mhamphi, G.G., Kazwala, R.R., 2015. Predominant leptospiral serogroups circulating among humans, livestock and wildlife in

Katavi-Rukwa ecosystem, Tanzania. PLoS Negl. Trop. Dis. 9. https://10.1371/journal.pntd.0003607.

Bahaman, A.R., Ibrahim, A.L., Adam, H., 1987. Serological Prevalence of Leptospiral Infection in Domestic Animals in West Malaysia. Epidemiol. Infec. 99, 379–392. https://10.1017/s0950268800067868.

Balamurugan, V., Alamuri, A., Kumar, K.V., Varghese, B., Govindaraj, G., Hemadri, D., Roy, P., 2021. Prevalence of Anti-Leptospiral Antibodies and Frequency Distribution of *Leptospira* Serovars in Small Ruminants in Enzootic South Peninsular India. Vet. World 14, 2023–2030. https://10.14202/vetworld.2021.2023-2030.

Balamurugan, V., Thirumalesh, S.R.A., Sridevi, R., Govindaraj, G., Nagalingam, M., Hemadri, D., Gajendragad, M. R., Rahman, H., 2016. Microscopic agglutination test analysis identifies prevalence of intermediate species serovars in ruminants in endemic states of India. Proceed. Nat. Acad. Sci. India Section B – Biol. Sci. 86, 469–475. https://10.1007/s40011-014-0469-6.

Balamurugan, V., Veena, S., Thirumalesh, S.R.A., Alamuri, A., Sridevi, R., Sengupta, P.P., Govindaraj, G., Nagalingam, M., Hemadri, D., Gajendragad, M.R., Rahman, H., 2017. Distribution of Serogroup Specific Antibodies against Leptospirosis in Livestock in Odisha. Indian J. Anim. Sci. 87, 546–551. http://10.56093/ijans.v87i5.70223.

Benkirane, A., Essamkaoui, S., El Idrissi, A., Lucchese, L., Natale, A., 2015. A sero-survey of major infectious causes of abortion in small ruminants in Morocco. Vet. Ital., 51, 25–30. https://10.12834/VetIt.389.1814.1.

Benkirane, A., Noury, S., Hartskeerl, R.A., Goris, M.G.A., Ahmed, A., Nally, J.E., 2016. Preliminary Investigations on the Distribution of *Leptospira* Serovars in Domestic Animals in North-west Morocco. Transbound. Emerg. Dis. 63, 178–184. https://10.1111/tbed.12252.

Bertelloni, F., Cilia, G., Turchi, B., Pinzauti, P., Cerri, D., Fratini, F., 2019. Epidemiology of Leptospirosis in North-Central Italy: Fifteen Years of Serological Data (2002–2016). Comp. Immunol. Microbiol. Infect. Dis. 65, 14–22. https://10.1016/j.cimid.2019.04.001.

Burriel, A.R., Dalley, C., Woodward, M.J., 2003. Prevalence of *Leptospira* Species among Farmed and Domestic Animals in Greece. Vet. Rec. 153, 146–148. https://10.1136/vr.153.5.146.

Campos, Â.P., Miranda, D.F.H., Rodrigues, H.W.S., Lustosa, M.S.C., Martins, G.H.C., Mineiro, A.L.B.B., Castro, V., Azevedo, S.S., Silva, S.M.M.S., 2017. Seroprevalence and Risk Factors for Leptospirosis in Cattle, Sheep, and Goats at Consorted Rearing from the

State of Piauí, Northeastern Brazil. Trop. Anim. Health Prod. 49, 899–907. https://10.1007/s11250-017-1255-2.

Carvalho, R.R.M., Paz, L.N., Dias, C.S., Nocera, G.A., Mesquita, A.J.P., Pinna, M.H., 2021. Serological survey of leptospirosis, brucellosis, and lentivirus in herds of small ruminants in Recôncavo Baiano, Bahia, Brazil. Braz. J. Vet. Res. Anim. Sci. 58. https://10.11606/issn.1678-4456.bjvras.2021.180290.

Ciceroni, L., Lombardo, D., Pinto, A., Ciarrocchi, S., Simeoni, J., 2000. Prevalence of Antibodies to *Leptospira* Serovars in Sheep and Goats in Alto Adige-South Tyrol. J. Vet. Med. B. Infect. Dis. Vet. Public Health 47, 217–223. https://10.1046/j.1439-0450.2000.00333.x.

Cortizo, P., Loureiro, A.P., Martins, G., Rodrigues, P.R., Faria, B.P., Lilenbaum, W., Deminicis, B.B., 2015. Risk factors to incidental leptospirosis and its role on the reproduction of ewes and goats of Espírito Santo state, Brazil. Trop. Anim. Health Prod. 47, 231–235. https://10.1007/s11250-014-0684-4.

Costa, D.F., Silva, A.F., Farias, A.E.M., Brasil, A.W.L., Santos, F.A., Guilherme, R.F., Azevedo, S.S., Alves, C.J., 2016. Serological study of the *Leptospira* spp. infection in sheep and goats slaughtered in the State of Paraíba, semiarid of Northeastern Brazil. Semina: Ciências Agrárias 37, 819–828. https://10.5433/1679-0359.2016v37n2p819.

Cranford, H.M., Taylor, M., Browne, A.S., Alt, D.P., Anderson, T., Hamond, C., Hornsby, R.L., LeCount, K., Schlater, L., Stuber, T., Wilde, L., Burke-France, V.J., Ellis, E.M., Nally, J.E., Bradford, B., 2021. Exposure and Carriage of Pathogenic *Leptospira* in Livestock in St. Croix, U.S. Virgin Islands. Trop. Med. Infec. Dis. 6, 85. https://10.3390/tropicalmed6020085.

Crespo, E., García, A., Rivero, J., Gómez, A., 2013. Seroprevalencia de leptospirosis en cabras de la parroquia Faría, municipio Miranda, estado Zulia-Venezuela. Rev. Cient. FCV-LUZ 23, 287–292. https://www.redalyc.org/articulo.oa?id=95926991003.

Czopowicz, M., Kaba, J., Smith, L., Szalus-Jordanow, O., Nowicki, M., Witkowski, L., Frymus, T., 2011. Leptospiral Antibodies in the Breeding Goat Population of Poland. Vet. Rec. 169, 230. https://10.1136/vr.d4403.

Damude, D.F., Jones, C.J., Myers, D.M., 1979. Study of Leptospirosis among Animals in Barbados W.I. Trans. R. Soc. Trop. Med. Hyg. 73, 161–168. https://10.1016/0035-9203(79)90200-1.

Desvars A., Naze F., Benneveau, A., Cardinale, E., Michault, A., 2013. Endemicity of leptospirosis in domestic and wild animal species from Reunion Island (Indian Ocean). Epidemiol. Infect. 141, 1154-65. https://10.1017/S0950268812002075.

Dhivahar, M., Ambily, R., Joseph, S., Shyma, V.H., Reshma, P.S., Mini, M., 2019. Seroprevalence of Leptospirosis among Aborted Goats in Kerala. Int. J. Curr. Microbiol. App. Sci. 8, 1403–1407. https://10.20546/ijcmas.2019.808.163.

Everard, C. O., Fraser-Chanpong, G. M., James, A. C., Butcher, L. V., 1985. Serological studies on leptospirosis in livestock and chickens from Grenada and Trinidad. Trans. R. Soc. Trop. Med. Hyg. 79, 859–864. https://10.1016/0035-9203(85)90138-5.

Galvão, C.M.M.Q., Oliveira, P.R.F., Cavalcanti, A.L.A., Nogueira, D.B., Azevedo, S.S., Ramos, R.A.N., Mota, R.A., 2023. Occurrence of IgG antibodies against *Toxoplasma gondii, Neospora caninum,* and *Leptospira* spp. in goats and sheep from an indigenous village in Pernambuco. Braz. J. Vet. Paras. 32. https://doi.org/10.1590/S1984-29612023022.

Gaytán-Camarillo, F., Rico-Chávez, O., Palomares-Resendiz, E.G., Gutiérrez-Hernández, J.L., Díaz-Aparicio, E., Herrera-López, E., 2021. Spatial Autocorrelation and Co-Occurrence of Six Serovarieties of *Leptospira* in Goat Herds of the State of Guanajuato, Mexico. Braz. J. Microbiol. 52, 953–960. https://10.1007/s42770-021-00429-y.

Guzman-Barragan, B.L., Martínez-Rodríguez, L.C., Tobón-Torreglosa, J.C., Tafur-Gómez, G.A., 2022. Seroprevalence and Risk Factors for *Leptospira* spp. in Small Ruminants of Semi-Arid Zone in Northeastern Colombia. Trop. Anim. Health Prod. 54. https://10.1007/s11250-021-03019-0.

Hamond, C., Martins, G., Loureiro, A.P., Pestana, C., Lawson-Ferreira, R., Medeiros, M.A., Lilenbaum, W., 2014. Urinary PCR as an increasingly useful tool for an accurate diagnosis of leptospirosis in livestock. Vet. Res. Comm. 38, 81–85. https://10.1007/s11259-013-9582-x.

Hassanpour, A., Asgarloo, S., Imandar, M., Mashayekhi, M., Abdollahpour, G.R., Safarmashaei, S., 2012. Seroepidemiologic study of goats leptospirosis in Khoy-Iran. J. Anim. Vet. Adv. 11, 229–233. https://10.3923/javaa.2012.229.233.

Higino, S.S., Alves, C.J., Santos, C.S.A.B., Vasconcellos, S.A., Silva, M.L.C.R., Brasil, A.W.L., Pimenta, C.L.R.M., Azevedo, S.S., 2012. Prevalência de leptospirose em caprinos leiteiros do semiárido paraibano. Pesq. Vet. Bras. 32, 199–203. https://10.1590/S0100-736X2012000300003.

Krawczyk, M., 2005. Serological Evidence of Leptospirosis in Animals in Northern Poland. Vet. Rec. 156, 88–89. https://10.1136/vr.156.3.88.

Krupakaran, R.P., Porcheziyan, T., Sivseelan, S., 2009. Seroprevalence of Leptospirosis in Domestic Animals of Karur District of Tamil Nadu. Vet. Pract. 10, 84-85. https://typeset.io/papers/seroprevalence-of-leptospirosis-in-domestic-animals-of-karur-47hd1yq6pw.

Lilenbaum, W., Souza, G.N., Ristow, P., Moreira, M.C., Fráguas, S., Cardoso, V.S., Oelemann, W.M.R.A., 2007. A serological study on *Brucella abortus*, caprine arthritis-encephalitis virus and *Leptospira* in dairy goats in Rio de Janeiro, Brazil. Vet. J. 173, 408–412. https://10.1016/j.tvjl.2005.12.003.

Lilenbaum, W., Varges, R., Medeiros, L., Cordeiro, A.G., Cavalcanti, A., Souza, G.N., Richtzenhain, L., Vasconcellos, S.A., 2008. Risk factors associated with leptospirosis in dairy goats under tropical conditions in Brazil. Res. Vet. Sci. 84, 14–17. https://10.1016/j.rvsc.2007.03.011.

Maleki, S., Zakian, A., Abdollahpour, G., 2021. Seroepidemiology of *leptospira interrogans* infection in ruminants of Lorestan Province: A cross-sectional study. J. Vet. Res. 75, 486–497. https://10.22059/JVR.2019.269334.2869.

Martins, G., Penna, B., Hamond, C., Leite, R. C-K., Silva, A., Ferreira, A., Brandão, F., Oliveira, F., Lilenbaum, W., 2012. Leptospirosis as the most frequent infectious disease impairing productivity in small ruminants in Rio de Janeiro, Brazil. Trop. Anim. Health Prod. 44, 773–777. https://10.1007/s11250-011-9964-4.

Mgode, G.F., Mhamphi, G.G., Massawe, A.W., Machang'u, R.S., 2021. *Leptospira* Seropositivity in Humans, Livestock and Wild Animals in a Semi-Arid Area of Tanzania. Pathog. 10, 696. https://10.3390/pathogens10060696.

Motie, A., Myers, D.M., 1986. Leptospirosis in Sheep and Goats in Guyana. Trop. Anim. Health Prod. 18, 113–114. https://10.1007/BF02359724.

Natarajaseenivasan, K., Ratnam, S., 1999. Isolation of *Leptospira javanica* from sheep. Indian J. Anim. Sci. 69, 759–761. https://epubs.icar.org.in/index.php/IJAnS/article/view/21277.

Paramasivam, A., Jagatheesan, P.R., Reetha, T.L., Henry, A.C.E., 2014. Sero-prevalence of leptospirosis in small ruminants in Pudukkottai District of Tamil Nadu. Indian J. Vet. Sci. Biotechnol. 10, 23–24. https://acspublisher.com/journals/index.php/ijvsbt/article/view/2972.

Parveen, S.M.A., Suganyaa, B., Sathya, M.S., Margreat, A.A.P., Sivasankari, K., Shanmughapriya, S., Hoffman, N.E., Natarajaseenivasan, K., 2016. Leptospirosis

Seroprevalence Among Blue Metal Mine Workers of Tamil Nadu, India. Amer. J. Trop. Med. Hyg. 95, 38-42. https://10.4269/ajtmh.16-0095.

Pasquali, A.K.S., Chideroli, R.T., Benitez, A.N., Caldart, E.T., Evers, F., Fortes, M. S., Ferreira, F.P., Monterio, K. C., Giordano, L.G.P., Freire., L.R., Freitas, J.C., Navarro, I.T., 2017. Cross-sectional study of *Leptospira* spp. and *Brucela abortus* in goat herds from Paraná State, Brazil. Act. Sci. Vet. 45, 1-9. https://10.22456/1679-9216.79794.

Pimenta, C.L.R.M., Bezerra, C.S., Morais, D.A., Silva, M.L.C.R., Nogueira, D.B., Costa, D.F., Santos, C.S.A.B., Higino, S.S.S., Alves, C.J.; Azevedo, S.S., 2019. Seroprevalence and predominant serogroups of *Leptospira* sp. in serological tests of ruminants in Northeastern Brazil. Semina: Ciências Agrárias 40, 1513–1522. https://10.5433/1679-0359.2019v40n4p1513.

Rahman, M.S.A., Bejo, S.K., Zakaria, Z., Hassan, L., Roslan, M.A., 2021. Seroprevalence and distribution of Leptospiral Serovars in livestock (cattle, goats, and sheep) in Flood-Prone Kelantan, Malaysia. J. Vet. Res. 65, 53–58. https://10.2478/jvetres-2021-0003.

Ramin, A., Abdollahpour, G., Hosseinzadeh, A., Azizzadeh F., Ramin P., Klalili Y., Sanajo D., Nezhad S. I., 2023. Comparison of anti-*Leptospira* antibodies by microscopic agglutination test in ruminants and equines of Urmia, Iran. Vet. Res. Forum 14, 229–235. https://10.30466/vrf.2022.546475.3345.

Rezaei, S., Haji, H.M., Ghadrdan, M.A., Ghorbanpour, M., Abdollahpour, G., 2016. Comparison of *Leptospira interrogans* infection in the goats and sheep. Iranian J. Vet. Med. 10, 113-119. https://www.cabdirect.org/cabdirect/abstract/20163241128.

Rizzo, H., Silva, T.R., Carvalho, J.S., Marinho, F.A., Santos, H.A., Júnior, W.S.S., Alemán, M.A.R., Pinheiro Júnior, J.W., Castro, V., 2017. Frequency of and risk factors associated to *Leptospira* spp. Seropositivity in goats in the state of Sergipe, Northeastern Brazil. Ciência Rural, 47, e20160845. https://10.1590/0103-8478cr20160845.

Rocha, L.M.S.R., Faria, P.J.A., Soares, R.R., Araújo Júnior, J.P., Malossi, C.D., Ullmann, L.S., Silva, M.L.C.R., Higino, S.S.S., Azevedo, S.S., Alves, C.J., 2022. *Leptospira* spp. of the Urinary Tract of Female Carrier Goats in Semi-Arid Conditions. Act. Sci. Vet. 50, 1872. https://10.22456/1679-9216.124079.

Roqueplo, C., Kodjo, A., Demoncheaux, J.-P.; Scandola, P., Bassene, H., Diatta, G., Sokhna, C., Raoult, D., Davoust, B., Mediannikov, O., 2019. Leptospirosis, one neglected disease in rural Senegal. Vet. Med. Sci. 5, 536–544. https://10.1002/vms3.186.

Sabarinath, T., Behera, S.K., Deneke, Y., Atif Ali, S., Kaur, G., Kumar, A., Kumar, G.R., Kumar, K.S., Sinha, D.K., Verma, M.R., Srivastava, S.K., Chaudhuri, P., 2018.

Serological evidence of anti-*Leptospira* antibodies in goats in various agro climatic zones of India. Small Ruminant Res. 169, 74–80. https://10.1016/j.smallrumres.2018.10.013.

Santos, J.P., Lima-Ribeiro, A.M.C., Oliveira, P.R., Santos, M.P., Ferreira Júnior, Á., Medeiros, A.A., Tavares, T.C.F., 2012. Seroprevalence and risk factors for Leptospirosis in goats in Uberlândia, Minas Gerais, Brazil. Trop. Anim. Health Prod. 44, 101–106. https://10.1007/s11250-011-9894-1.

Santos, L.F., Guimarães, M.F., Souza, G.O., Silva, I.W.G., Santos, J.R., Azevedo, S.S., Labruna, M.B., Heinemann, M.B., Horta, M.C., 2017. Seroepidemiological survey on *Leptospira* spp. infection in wild and domestic mammals in two distinct areas of the semi-arid region of northeastern Brazil. Trop. Anim. Health Prod. 49, 1715–1722. https://10.1007/s11250-017-1382-9.

Saranya, P., Goswami, C., Sumathi, K., Balasundareshwaran, A.H., Bothammal, P., Dutta, L.J., Muralitharan, G., Bora, D.P., Natarajaseenivasan, K., 2021. Prevalence of leptospirosis among animal herds of northeastern provinces of India. Comp. Immunol. Microbiol. Infect. Dis. 79, 101698. https://10.1016/j.cimid.2021.101698.

Schmidt, V., Arosi, A., Santos, A.R., 2002. Levantamento sorológico da leptospirose em caprinos leiteiros no Rio Grande do Sul, Brasil. Ciência Rural 32, 609–12. https://10.1590/S0103-84782002000400010.

Schnydrig, P., Vidal, S., Brodard, I., Frey, C., Posthaus, H., Perreten, V., Rodriguez-Campos, S., 2017. Bacterial, fungal, parasitological and pathological analyses of abortions in small ruminants from 2012-2016. Schweiz. Arch. Tierheilkd 159, 647-656. https://10.17236/sat00136.

Shigidi, M.T.A., 1974. Animal Leptospirosis in the Sudan. Brit. Vet. J. 130, 528–531. https://10.1016/S0007-1935(17)35738-X.

Shiokawa, K., Welcome, S., Kenig, M., Lim, B., Rajeev, S., 2019. Epidemiology of *Leptospira* infection in livestock species in Saint Kitts. Trop. Anim. Health Prod. 51, 1645–1650. https://10.1007/s11250-019-01859-5.

Shrestha, R., McKenzie, J.S., Gautam, M., Adhikary, R., Pandey, K., Koirala, P., Bahadur B.C.G., Laurie C. Miller., Collins-Emerson, J., Craig, S.B., Shrestha, S., Heuer, C., 2018. Determinants of clinical leptospirosis in Nepal. Zoonoses Public Health 65, 972–983. https://10.1111/zph.12516.

Silva, F.J., Santos, C.E.P., Silva, T.R., Silva, G.C.P., Loffler, S.G., Brihuega, B., Alarcon, M.F.F., Curci, V.C.M., Mathias, L.A., 2015. Search of leptospires and of antibodies against leptospires in animals and human beings in farms in Pantanal and Caatinga

Brazilian biomes. Braz. J. Vet. Res. Anim. Sci. 52, 234-248. https://10.11606/issn.1678-4456.v52i3p234-248.

Smith, C.E., Turner, L.H., Harrison, J.L., Broom, J.C., 1961. Animal leptospirosis in Malaya: 1. Methods, zoogeographical background, and broad analysis of results. Bull. World Health Organ. 24, 5–21. https://pubmed.ncbi.nlm.nih.gov/20604085/.

Soares, R.R., Barnabé N.N.C., Araújo Júnior J.P., Malossi, C.D., Ullmann, L.S., Costa, D.F., Silva, M.L.C.R., Higino, S.S.S., Azevedo, S.S., Alves, C.J., 2023. Investigation of the Presence of *Leptospira interrogans* in Urinary and Genital Tracts of Male Goats Raised in the Semiarid Region of Brazil. Small Ruminant Res. 218, 106880. https://doi.org/10.1016/j.smallrumres.2022.106880.

Soares, R.R., Barnabé N.N.C., Silva, M.L.C.R., Costa, D.F., Araújo Júnior, J.P.A., Malossi, C.D., Ullmann, L.S., Higino, S.S.S., Azevedo, S.S., Alves, C.J., 2022. Detection of *Leptospira* spp. in genitourinary tract of female goats managed in the brazilian semiarid. Microbial Pathog. 172, 105763. https://10.1016/j.micpath.2022.105763.

Souza, M.A., Castro, J.R., Moreira, R.Q., Bombonato, N.G., Soares, P.M., Lima, A.M.C., 2016. Anti-*Leptospira* spp. antibodies in several animal species on the same farm. Biosci. J. 32, 202-207. https://10.14393/BJ-v32n1a2016-26605.

Suarez, V.H., Martínez, G.M., Olmos, L.H., Arapa, C., Cortez, H.S., Rojas, M.C., Brihuega, B.F., Santillán, G., Álvarez, I., Goz, M.L., 2020. Problemas sanitarios de las majadas caprinas en los sistemas familiares de los valles calchaquies (Payogasta, Salta). FAVE, Secc. Cienc. Vet. 19, 40–49. https://doi.org/10.14409/favecv.v19i2.9507.

Suepaul, S.M., Carrington, C.V., Campbell, M., Borde, G., Adesiyun, A.A., 2011. Seroepidemiology of leptospirosis in livestock in Trinidad. Trop. Anim. Health Prod. 43, 367–375. https://10.1007/s11250-010-9698-8.

Sunder, J., Rai, R.B., Kundu, A., Chatterjee, R. N., Senani, S., Jeyakumar, S., 2005. Incidence and prevalence of livestock diseases of Andaman and Nicobar Islands. Indian J. Anim. Sci.75, 1041-1043.https://scholar.google.com/citations?user=VrHbXCoAAAAJ&hl=en.

Suwancharoen, D., Chaisakdanugull, Y., Thanapongtharm, W., Yoshida, S., 2013. Serological survey of leptospirosis in livestock in Thailand. Epidemiol. Infect. 14, 2269–2277. https://10.1017/S0950268812002981.

Tagliabue, S., Figarolli, B.M., D'Incau, M., Foschi, G., Gennero, M.S., Giordani, R., Giordani, R., Natale, A., Papa, P., Ponti, N., Scaltrito, D., Spadari, L., Vesco, G., Ruocco, L., 2016. Serological surveillance of Leptospirosis in Italy: two-year national data (2010-2011). Vet. Ital. 52, 129-38. https://10.12834/VetIt.58.169.2.

Topazio, J., Tonin, A.A., Machado, G., Noll, J.C.G., Ribeiro, A., Moura, A.B., Carmo, G.M., Grosskopf, H.M., Martins, J.L.R., Badke, M.R.T., Stefani, L.M., Lopes, L.S., Silva, A.S., 2015. Antibodies to *Leptospira interrogans* in goats and risk factors of the disease in Santa Catarina (West side), Brazil. Res. Vet. Sci. 99, 53–57. https://10.1016/j.rvsc.2015.01.014.

Valeris-Chacín, R., Boscán-Duque, L., Urdaneta-Pacheco, R., Chango-Villasmil, J., Torres-Rodríguez, P., Quíntero-Moreno, A., Arzalluz-Fischer, A., Sánchez-Villalobos, A., 2012. Seroprevalence of Leptospirosis and Brucellosis in Goat Farms from Mauroa County, Falcon State, Venezuela. Rev. Cient. FCV-LUZ 22, 231–237. https<https://www.redalyc.org/pdf/959/95922219006.pdf.

Viana, M.P., Silva, J.D., Lima, A.M.C., Alves, F.S.F., Pinheiro, R.R.; Costa, D.F.; Silva, G.C.P., Calado, L.G.L.P., Azevedo, S.S., Alves, C.J., 2022. Epidemiological and geospatial characterization of goat leptospirosis in Northeast region of Brazil. Small Ruminant Res., 206, 106589. https://10.1016/j.smallrumres.2021.106589.

Vihol, P.D., Patel, J.M., Patel, J.H., Prasad, M.C., Kalyani, I.H., Brahmkshtri, B.P., 2016. Caprine leptospirosis: Hematobiochemical and urinalyses studies. Vet. World 9, 337–341. https://10.14202/vetworld.2016.337-341.

Vihol, P.D., Patel, J.M., Patel, J.H, Dabas, V.S., Kalyani, I.H., Chaudhari, C.F., Patel, A.C., 2017. Identification of Pathogenic *Leptospira* spp. Carrier Goats Using Polymerase Chain Reaction (PCR). Int. J. Curr. Microbiol. App. Sci. 6, 2174-2183. https://doi.org/10.20546/ijcmas.2017.612.249.

Vihol, P.D., Patel, J.M., Patel, J.H., Raval, J.K., Varia, R.D., Makwana, P.M., 2020. Pathomorphological study on leptospirosis in slaughtered goats. Pharma Innovation J. 9, 84-87. https://www.thepharmajournal.com/archives/2020/vol9issue9S/PartB/S-9-9-17-981.pdf
