# Supplementary material for: Insights on the Pooled Prevalence and Global Distribution of Leptospirosis in Goats: Systematic Review and Meta-Analysis
Source: Microorganisms. 2024 Nov 22;12(12):2391. doi: 10.3390/microorganisms12122391 (PMC11676935; doi:10.3390/microorganisms12122391)
Supplement: Supplementary file 1 [file microorganisms-12-02391-s001.zip › microorganisms-3288296-table-S2.pdf]

Table S2 - Results of the critical assessment of the methodological quality of the studies included in the systematic review with meta-analysis on the global seroprevalence of leptospirosis in goats, using the critical assessment tool for studies with JBI prevalence data.

Article	Q1	Q2	Q3	Q4	Q5	Q6	Q7	Q8	Score	Risk of Bias
**Study 1**	U	U	Y	N	Y	Y	Y	N	4	Moderate
**Study 2**	U	N	Y	U	Y	Y	Y	N	4	Moderate
**Study 3**	Y	Y	Y	Y	Y	Y	Y	N	7	Low
**Study 4**	N	Y	Y	U	Y	Y	Y	N	5	Moderate
**Study 5**	Y	Y	Y	Y	Y	Y	Y	Y	8	Low
**Study 6**	N	Y	Y	Y	Y	Y	Y	N	6	Moderate
**Study 7**	U	Y	Y	U	Y	Y	Y	Y	6	Moderate
**Study 8**	N	U	N	U	Y	Y	Y	Y	4	Moderate
**Study 9**	U	Y	Y	U	Y	Y	Y	Y	6	Moderate
**Study 10**	Y	U	Y	U	Y	Y	Y	Y	6	Moderate
**Study 11**	N	N	N	Y	Y	Y	Y	Y	5	Moderate
**Study 12**	Y	N	Y	U	Y	Y	Y	Y	6	Moderate
**Study 13**	Y	U	Y	Y	Y	Y	Y	Y	7	Low
**Study 14**	U	Y	Y	U	Y	Y	Y	U	5	Moderate
**Study 15**	Y	U	Y	U	Y	Y	Y	Y	6	Moderate
**Study 16**	U	Y	Y	U	Y	Y	Y	Y	6	Moderate
**Study 17**	U	Y	Y	U	Y	Y	Y	U	5	Moderate
**Study 18**	Y	N	Y	U	Y	Y	Y	U	5	Moderate
**Study 19**	U	U	Y	N	Y	Y	Y	U	4	Moderate
**Study 20**	U	U	Y	N	Y	Y	Y	U	4	Moderate
**Study 21**	N	U	Y	U	Y	Y	Y	N	4	Moderate
**Study 22**	U	U	Y	N	Y	Y	Y	N	4	Moderate
**Study 23**	U	N	Y	U	Y	Y	Y	Y	5	Moderate
**Study 24**	U	Y	Y	U	Y	Y	Y	Y	6	Moderate
**Study 25**	N	Y	Y	Y	Y	Y	Y	N	6	Moderate
**Study 26**	N	U	Y	U	Y	Y	Y	N	4	Moderate
**Study 27**	U	Y	Y	U	Y	Y	Y	Y	6	Moderate
**Study 28**	Y	N	Y	Y	Y	Y	Y	Y	7	Low
**Study 29**	U	Y	Y	U	Y	Y	Y	Y	6	Moderate
**Study 30**	U	Y	Y	U	Y	Y	Y	Y	6	Moderate
**Study 31**	Y	Y	Y	Y	Y	Y	Y	Y	8	Low
**Study 32**	Y	Y	Y	Y	Y	Y	Y	N	7	Low
**Study 33**	U	U	Y	U	Y	Y	Y	N	4	Moderate
**Study 34**	N	U	N	U	Y	Y	Y	Y	4	Moderate
**Study 35**	Y	Y	Y	Y	Y	Y	Y	N	7	Low
**Study 36**	U	Y	Y	N	Y	Y	Y	Y	6	Moderate
**Study 37**	U	U	N	U	Y	Y	Y	N	3	High
**Study 38**	Y	Y	Y	Y	Y	Y	Y	Y	8	Low
**Study 39**	U	U	N	N	Y	Y	Y	N	3	High
**Study 40**	U	U	Y	U	Y	Y	Y	N	4	Moderate
**Study 41**	U	U	N	N	Y	Y	Y	N	3	High
**Study 42**	U	U	Y	N	Y	Y	Y	N	4	Moderate

**Study 43**	U	Y	Y	U	Y	Y	Y	N	5	Moderate
**Study 44**	U	Y	Y	U	Y	Y	Y	Y	6	Moderate
**Study 45**	N	N	Y	U	Y	Y	Y	N	4	Moderate
**Study 46**	N	Y	Y	Y	Y	Y	Y	N	6	Moderate
**Study 47**	U	U	N	U	Y	Y	Y	N	3	High
**Study 48**	Y	Y	Y	Y	Y	Y	Y	Y	8	Low
**Study 49**	Y	U	Y	Y	Y	Y	Y	N	6	Moderate
**Study 50**	N	U	Y	Y	Y	Y	Y	Y	6	Moderate
**Study 51**	N	U	Y	Y	Y	Y	Y	N	5	Moderate
**Study 52**	U	Y	Y	U	Y	Y	Y	N	5	Moderate
**Study 53**	U	Y	Y	U	Y	Y	Y	N	5	Moderate
**Study 54**	U	U	Y	N	Y	Y	Y	Y	5	Moderate
**Study 55**	U	Y	Y	N	Y	Y	Y	Y	6	Moderate
**Study 56**	Y	U	Y	Y	Y	Y	Y	N	6	Moderate
**Study 57**	N	Y	Y	Y	Y	Y	Y	Y	7	Low
**Study 58**	U	Y	Y	N	Y	Y	Y	Y	6	Moderate
**Study 59**	Y	N	Y	Y	Y	Y	Y	Y	7	Low
**Study 60**	Y	Y	Y	U	Y	Y	Y	Y	7	Low
**Study 61**	U	U	N	N	Y	Y	Y	Y	4	Moderate
**Study 62**	N	Y	Y	Y	Y	Y	Y	Y	7	Low
**Study 63**	U	N	Y	U	Y	Y	Y	N	4	Moderate
**Study 64**	U	Y	Y	U	Y	Y	Y	N	5	Moderate
**Study 65**	U	Y	Y	N	Y	Y	Y	N	5	Moderate
**Study 66**	U	Y	Y	N	Y	Y	Y	Y	6	Moderate
**Study 67**	U	Y	Y	Y	Y	Y	Y	Y	7	Low
**Study 68**	U	U	Y	N	Y	Y	Y	N	4	Moderate
**Study 69**	U	Y	Y	N	Y	Y	Y	N	5	Moderate
**Study 70**	U	Y	Y	N	Y	Y	Y	N	5	Moderate
**Study 71**	Y	U	Y	N	Y	Y	Y	N	5	Moderate
**Study 72**	U	U	Y	N	Y	Y	Y	N	4	Moderate
**Study 73**	U	U	Y	N	Y	Y	Y	N	4	Moderate
**Study 74**	N	U	Y	Y	Y	Y	Y	N	5	Moderate
**Study 75**	U	U	Y	N	Y	Y	Y	N	4	Moderate
**Study 76**	U	U	Y	N	Y	Y	Y	N	4	Moderate
**Study 77**	U	Y	N	N	Y	Y	Y	N	4	Moderate
**Study 78**	U	U	Y	N	Y	Y	Y	N	4	Moderate
**Study 79**	U	U	Y	N	Y	Y	Y	N	4	Moderate

Y- Yes, N- No, U- Uncertain
